# Apparent diffusion coefficient predicts MGMT status in adult-type diffuse gliomas and is correlated with Ki-67 proliferation index

**DOI:** 10.3389/fonc.2025.1609562

**Published:** 2025-09-01

**Authors:** Feng-Ying Zhu, Li-Yong Zhuo, Tian-Da Wang, Jia-Ning Wang

**Affiliations:** Department of Radiology, Affiliated Hospital of Hebei University, Baoding, China

**Keywords:** apparent diffusion coefficient, MGMT promoter methylation, IDH mutation, Ki-67, glioma

## Abstract

**Purpose:**

To evaluate the predictive value of apparent diffusion coefficient (ADC) for O6-methylguanine-DNA methyltransferase (MGMT) promoter methylation and its correlation with Ki-67 proliferation index in adult-type diffuse glioma, stratified by isocitrate dehydrogenase (IDH) subtype.

**Methods:**

This retrospective study enrolled 94 patients with pathologically confirmed glioma (2017–2024). ADCmin, ADCmean, and relative ADC (rADC) values were derived from diffusion-weighted imaging (b=1000 s/mm²). MGMT methylation, IDH mutation, and Ki-67 index were assessed by Pyrosequencing and immunohistochemistry. Receiver operating curve analysis was performed to evaluate diagnostic performance, and Spearman correlation was used to link ADC with Ki-67 index.

**Results:**

MGMT-methylated gliomas exhibited significantly higher ADCmin (0.86 vs. 0.74 × 10^-^³mm²/s, *p* = 0.013) and rADCmin (1.12 vs. 0.95, *p*<0.001). In the IDH-wild-type subgroup, rADCmin achieved an AUC of 0.78 (cutoff=1.10, sensitivity=80.0%). All ADC parameters were negatively correlated with Ki-67 (*ρ*=- 0.32 to -0.24, *p*<0.05).

**Conclusion:**

ADC values, particularly rADCmin, were identified as non-invasive biomarkers for MGMT methylation prediction in IDH-wild-type gliomas. An inverse correlation between ADC and Ki-67 index supported their utility for assessing tumor proliferation. Standardizing rADC would improve its clinical applicability across imaging platforms.

## 1 Introduction

Gliomas are the most common primary malignant tumors of the central nervous system (CNS), and their molecular heterogeneity affects treatment strategies and prognosis (1). The methylation status of the O6-methylguanine-DNA methyltransferase (MGMT) promoter is a key predictor of temozolomide chemosensitivity (2), whereas isocitrate dehydrogenase (IDH) mutations are strongly associated with molecular typing of gliomas and good prognosis (3). The 2021 WHO classification of CNS tumors emphasizes the clinical significance of molecular typing (4). Currently, the gold standard test for MGMT status relies on postoperative pathology, and noninvasive imaging biomarkers are urgently needed to improve the accuracy of pre-operative assessment.

Apparent diffusion coefficient (ADC) —a quantitative parameter derived from diffusion-weighted imaging (DWI)—reflects tumor cell density and microstructure by diffusion-weighted imaging (DWI) (5). Although ADC values can be used for glioma grading and IDH mutation prediction (6, 7), their utility for identifying the methylation status of MGMT promoters remains controversial (8). In addition, most studies are limited to single molecular marker analyses and lack standardized ADC values to reduce the influence of individual differences (9). In this study, we analyzed the efficacy of ADC values for determining MGMT status in conjunction with IDH typing stratification and examined the effect of rADC standardization for improving the generalizability of this method. This study also explored the correlation between ADC values and Ki-67 proliferation index, which provided a basis for clinical diagnosis and treatment.

## 2 Materials and methods

### 2.1 General information

**Figure 1:**
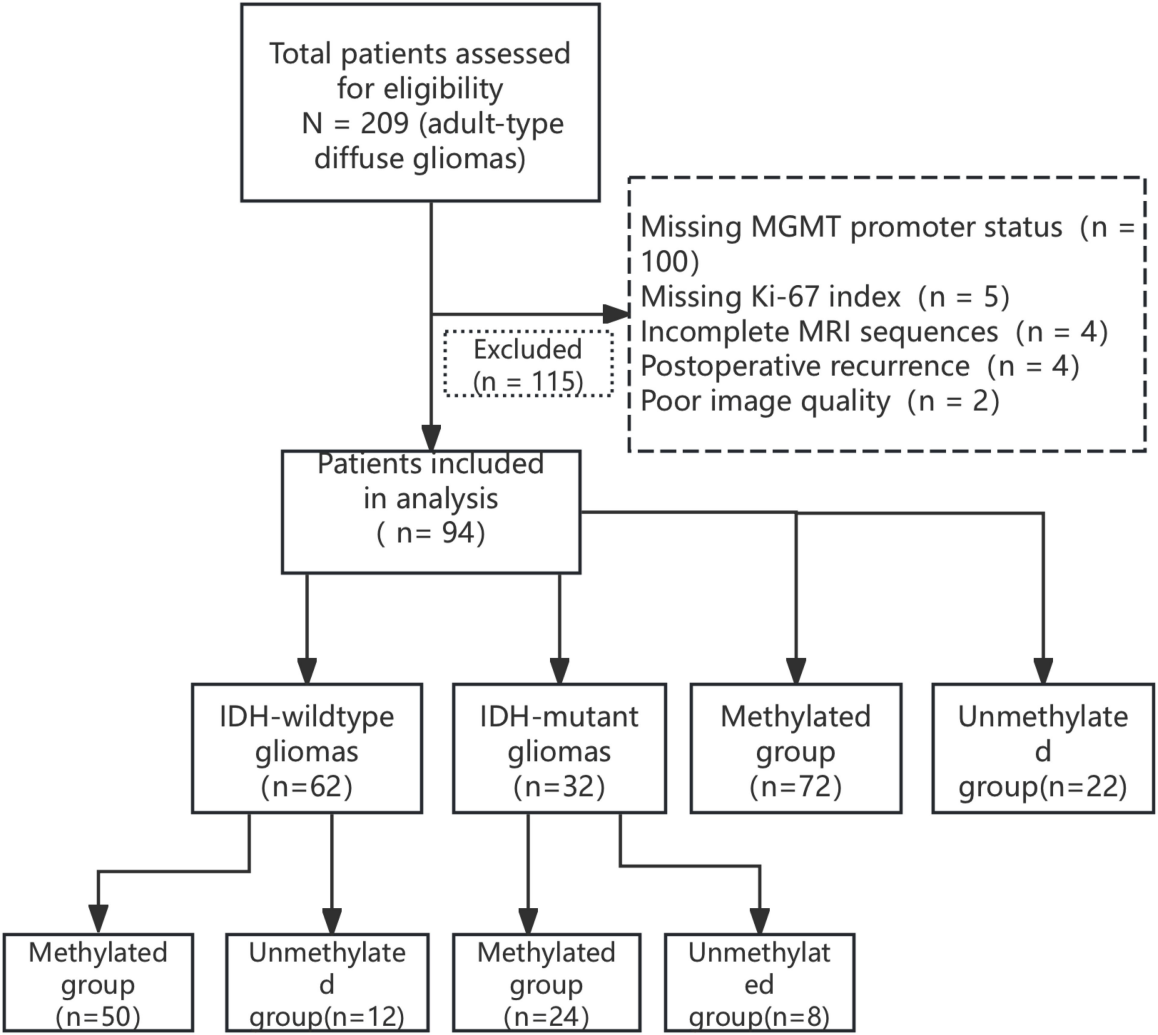
Flow diagram of the patient selection process.

This study was a retrospective analysis of 94 patients with adult-type diffuse glioma including 56 men and 38 women aged 43 – 76 (55.2 ± 11.7) years who were diagnosed pathologically in Hebei University Hospital between 2017 and 2024 (**Figure 1**). The inclusion criteria were as follows: ① complete MRI examination (including T1WI, T2WI, FLAIR, T1WI-CE, and DWI sequences) before surgery; ② pathologically confirmed diagnosis of adult-type diffuse glioma; ③ complete MGMT, IDH, and Ki-67 test data. The exclusion criteria were as follows: ① non-adult-type diffuse glioma pathologic type; ② poor image quality, image diagnosis, and data measurement; ③ missing clinical data. The study was approved by the Ethics Committee of the Affiliated Hospital of Hebei University (Grant No. HDFYLL-KY-2024-178), and the requirement for informed consent was waived by the Ethics Committee due to the retrospective nature of the study.

### 2.2 Image data acquisition and analysis

**Figure 2:**
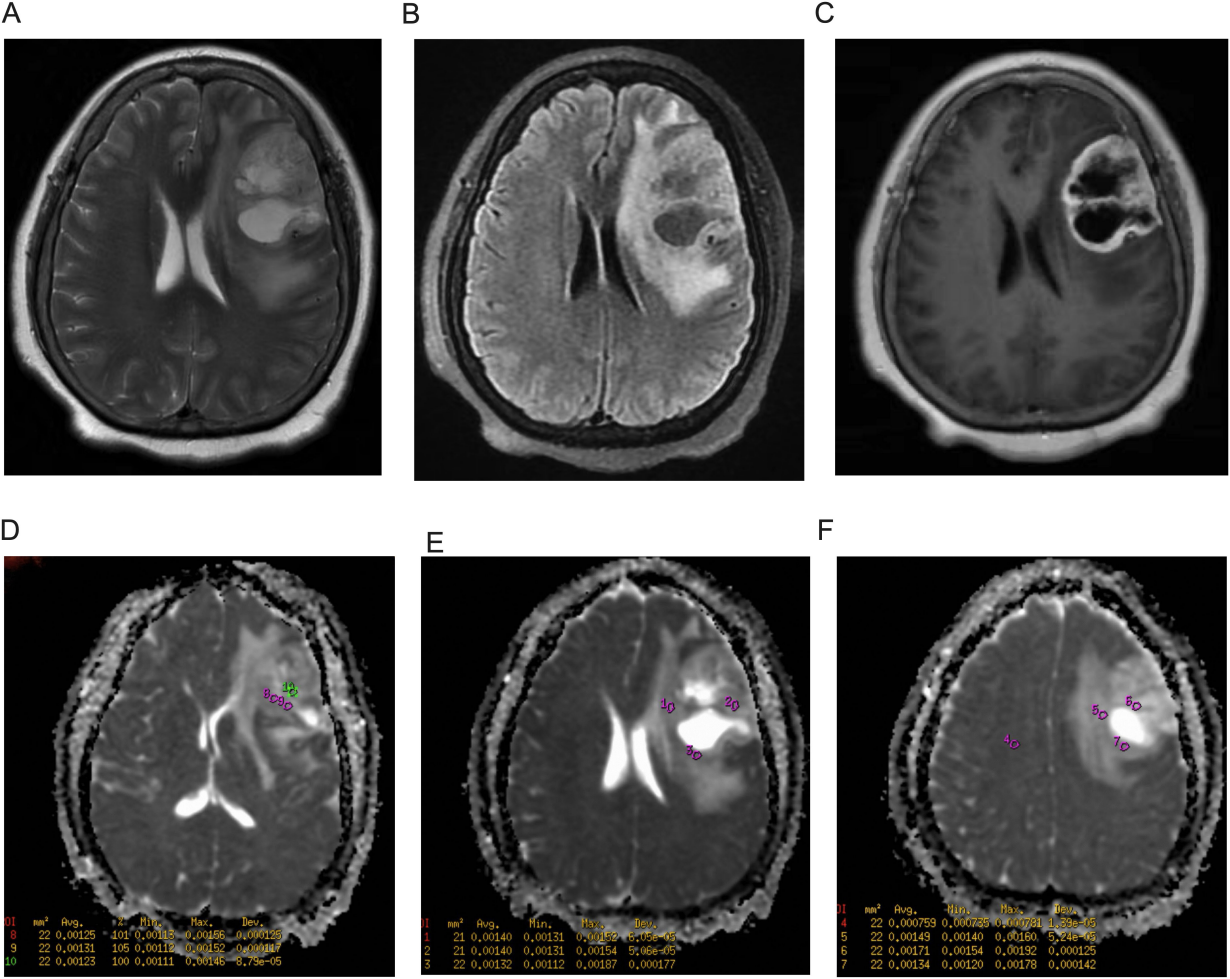
Region of Interest (ROI) Placement protocol for ADC quantification. **(A)** Axial T2-weighted image (T2WI) at maximal tumor cross-section. **(B)** Axial fluid-attenuated inversion recovery (FLAIR) image. **(C)** Axial contrast-enhanced T1-weighted image (T1WI-CE) delineating solid tumor enhancement. **(D-F)** Grayscale ADC maps with ROIs (color circles) at three tumor levels: **(D)** Slice with 3 ROIs; **(E)** Maximal cross-sectional slice with 3 ROIs (ADCmean = 1.37 × 10^-^³ mm²/s); **(F)** Slice with 3 ROIs + contralateral reference ROI (ROI 4). ADCmin (1.11 × 10^-^³ mm²/s) derived from the lowest value among all 9 tumor ROIs. All images cropped to remove peripheral space. Patient: 73M, IDH-wt glioblastoma.

A 3.0T MRI scanner (GE Discovery MR750) was used to acquire DWI sequences (b = 0, 1000 s/mm²) using the following acquisition parameters: slice thickness = 5 mm, TR = 2347 ms, TE = minimum, matrix size = 128 × 128, and field of view (FOV) = 240 × 240 mm. Raw images of the DWI sequences were imported for post-processing. Grayscale ADC maps were generated on the workstation. Two neuroradiologists (Wang JN and Zhu FY, with 14 and 8 years’ experience respectively) independently placed circular ROIs under blinding to clinical data. ROI placement was guided by T1WI-CE and FLAIR sequences to target solid enhancing tumor areas, while avoiding necrosis/cysts/hemorrhage (defined as T1-hypointense, T2/FLAIR-hyperintense regions). Three circular ROIs (15 – 25 mm²) were positioned at each of three consecutive axial slices (including the maximal cross-sectional slice), totaling nine ROIs per tumor. ADCmin was defined as the lowest value among all nine tumor ROIs. ADCmean was calculated as the average of the three ROIs at the maximal cross-sectional slice. An ROI of 15 – 25 mm² was placed in the contralateral semioval center to minimize partial volume effects while ensuring measurement stability. Relative values were computed as: rADCmin = ADCmin/normal WM ADC, rADCmean = ADCmean/normal WM ADC (**Figure 2**). Interobserver agreement was assessed using intraclass correlation coefficient (ICC) based on a two-way random-effects model for absolute agreement. We reported the average-measures ICC (ICC[A,1]) since the mean values of both readers were used in subsequent analyses. The ICC was calculated with 95% confidence intervals using SPSS 26.0 (Model 2, k=2 raters), with values >0.85 indicating excellent reliability.

### 2.3 Molecular pathology testing

The methylation level of the MGMT promoter CpG island was quantitatively analyzed using pyrosequencing, with a methylation threshold ≥8% defined as positive (10). Mutations in IDH1 R132H and IDH2 R172K sites were detected by Sanger sequencing, and primer sequences were obtained from the COSMIC database (11) (forward: 5′-TGTGTTGAGATGGGACGCCTA-3′; reverse: 5′ -TGCCAACATGACTTACTTGA-3′). Ki-67 proliferation index was determined by immunohistochemistry (antibody clone: MIB - 1, Dako, 1:100 dilution), and 10 randomly selected high magnification fields of view (×400) were used. The mean percentage of positive cells was recorded (12).

### 2.4 Statistical analysis

Statistical analyses were performed using SPSS 26.0; parameters that conformed to normal distribution were expressed as mean ± standard deviation, and differences between groups were compared using the independent samples t-test. Parameters that did not conform to normal distribution were expressed as median (third quartile-first quartile), and differences between groups were compared using the Mann-Whitney U-test. The performance of between-group differences in ADC min, ADC mean, rADC min, and rADC mean for differential diagnosis was assessed by receiver operating characteristic (ROC) curve. Correlations between ADC values and the Ki-67 proliferation index were analyzed using Spearman’s correlation coefficient. Differences with *p <*0.05 were considered statistically significant. Sample size was estimated based on previous studies and G*Power software; the settings were effect size *d* = 0.8, *α* = 0.05, and test efficacy 0.8, and estimating a total sample size of 58 cases. The study included 94 patients, fulfilling the minimum sample size requirement. For subgroup analyses (e.g., IDH mutant MGMT-negative group n = 10), nonparametric tests were used to reduce bias.

## 3 Results

### 3.1 General patient characteristics

Patient age was significantly higher in the MGMT unmethylated group (n = 22) than in the methylated group (n = 72) (65 vs. 57 years, *p* = 0.043), with no difference in gender distribution (*p* > 0.05). The age of MGMT unmethylated patients was higher than that of the methylated group in the IDH wild-type cohort (67 vs. 59 years, *p* = 0.021), whereas there was no statistically significant difference in the mutant group (**Table 1**), Among the 22 IDH-mutant gliomas, 1p19q status was available for 12 cases: 3 with intact 1p/19q, 8 with 1p/19q codeletion, and 1 with isolated 19q deletion. Statistical comparison of ADC values across these subgroups was not performed due to limited sample size and heterogeneity.

**Table 1:** Baseline characteristics of patients (n=94).

Group	MGMT Status		IDH Wild-Type		IDH Mutant-Type	
	MGMT (+)	MGMT (-)	MGMT (+)	MGMT (-)	MGMT (+)	MGMT (-)
Cases (n)	72	22	50	12	22	10
Age (years)	57(48 - 65)	65(59 - 70)	59(50 - 66)	67(63 - 72)	51(43 - 58)	53(46 - 58)
Male (%)	49 (68.06)	12 (54.55)	35 (70.00)	6 (50.00)	16 (72.73)	6 (60.00)
Female (%)	23 (31.94)	10 (45.45)	15 (30.00)	6 (50.00)	6 (27.27)	4 (40.00)
*p (*Age)	0.043*		0.021*		0.610	
*p (Sex*)	0.245		0.186		0.682	

Bold values indicate statistically significant p-values (p < 0.05).

In our study cohort, the distribution of WHO grades among the gliomas was as follows: 21 cases were WHO grade 2, 20 cases were WHO grade 3, and 53 cases were WHO grade 4. This distribution reflects the histological diversity of the tumors included in our analysis.

### 3.2 Consistency between two observers

Consistency of ADC measurements between the two physicians was determined by calculating the ICC. The results showed an ICC = 0.89 [95% confidence interval (CI): 0.82 – 0.93] for ADCmin and ICC = 0.85 (95% CI: 0.78 – 0.90) for ADCmean, showing good agreement.

### 3.3 Between-group differences in ADC indicators

In the comparison of the methylation status of the MGMT promoter, the methylation group showed a significant increase in ADCmin (0.86 ± 0.15 vs. 0.74 ± 0.12 × 10^-^³ mm²/s, *p* = 0.013) and ADCmean (1.12 ± 0.21 vs. 1.03 ± 0.18 × 10^-^³ mm²/s, *p* = 0.021), and relative values (rADCmin: 1.12 ± 0.15 vs. 0.95 ± 0.10, *p* < 0.001; rADCmean: 1.42 ± 0.25 vs. 1.25 ± 0.20, *p* = 0.005) were significantly higher than those of the unmethylated group. IDH subgroup analysis showed that in IDH wild-type gliomas, the MGMT promoter-methylated group exhibited significantly higher values than the unmethylated group in ADCmin (0.84 ± 0.14 vs. 0.72 ± 0.11 ×10^-^³ mm²/s, *p* = 0.018), ADCmean (1.11 ± 0.18 vs. 0.98 ± 0.15 × 10^-^³ mm²/s, *p* = 0.021), rADCmin (1.10 ± 0.13 vs. 0.93 ± 0.09, *p* < 0.001), and rADCmean (1.38 ± 0.22 vs. 1.20 ± 0.18 × 10^-^³ mm²/s, *p* = 0.007). In the IDH mutant type group, ADC values did not differ significantly between groups (*p* > 0.05) (**Table 2**).

**Table 2:** Comparison of ADC values between groups.

Group	Parameter	MGMT (-)	MGMT (+)	*p Value*
MGMT (-) vs (+)	ADCmin (× 10^-^³ mm²/s)	0.74 ± 0.12	0.86 ± 0.15	0.013*
	ADCmean (× 10^-^³ mm²/s)	1.03 ± 0.18	1.12 ± 0.21	0.021*
	rADCmin	0.95 ± 0.10	1.12 ± 0.15	<0.001*
	rADCmean	1.25 ± 0.20	1.42 ± 0.25	0.005*
IDH Wild-type MGMT (-) vs (+)	ADCmin (× 10³ mm²/s)	0.72 ± 0.11	0.84 ± 0.14	0.018*
	ADCmean (× 10^-^³ mm²/s)	0.98 ± 0.15	1.11 ± 0.18	0.021*
	rADCmin	0.93 ± 0.09	1.10 ± 0.13	<0.001*
	rADCmean	1.20 ± 0.18	1.38 ± 0.22	0.007*
IDH Mutant-Type MGMT (-) vs (+)	ADCmin (× 10^-^³ mm²/s)	0.78 ± 0.13	0.80 ± 0.16	0.610
	ADCmean (× 10^-^³ mm²/s)	1.30 ± 0.22	1.28 ± 0.20	0.610
	rADCmin	1.05 ± 0.12	1.08 ± 0.14	0.450
	rADCmean	1.35 ± 0.23	1.40 ± 0.21	0.320

Data are presented as mean ± SD. *p < 0.05 indicates statistical significance; ADCmin, minimum apparent diffusion coefficient; ADCmean, mean apparent diffusion coefficient; rADCmin, relative ADCmin (normalized to contralateral normal white matter); rADCmean, relative ADCmean; Non-normally distributed parameters (ADCmin, rADCmin) were analyzed using Mann-Whitney U test. Bold values indicate statistically significant p-values (p < 0.05).

**Figure 3:**
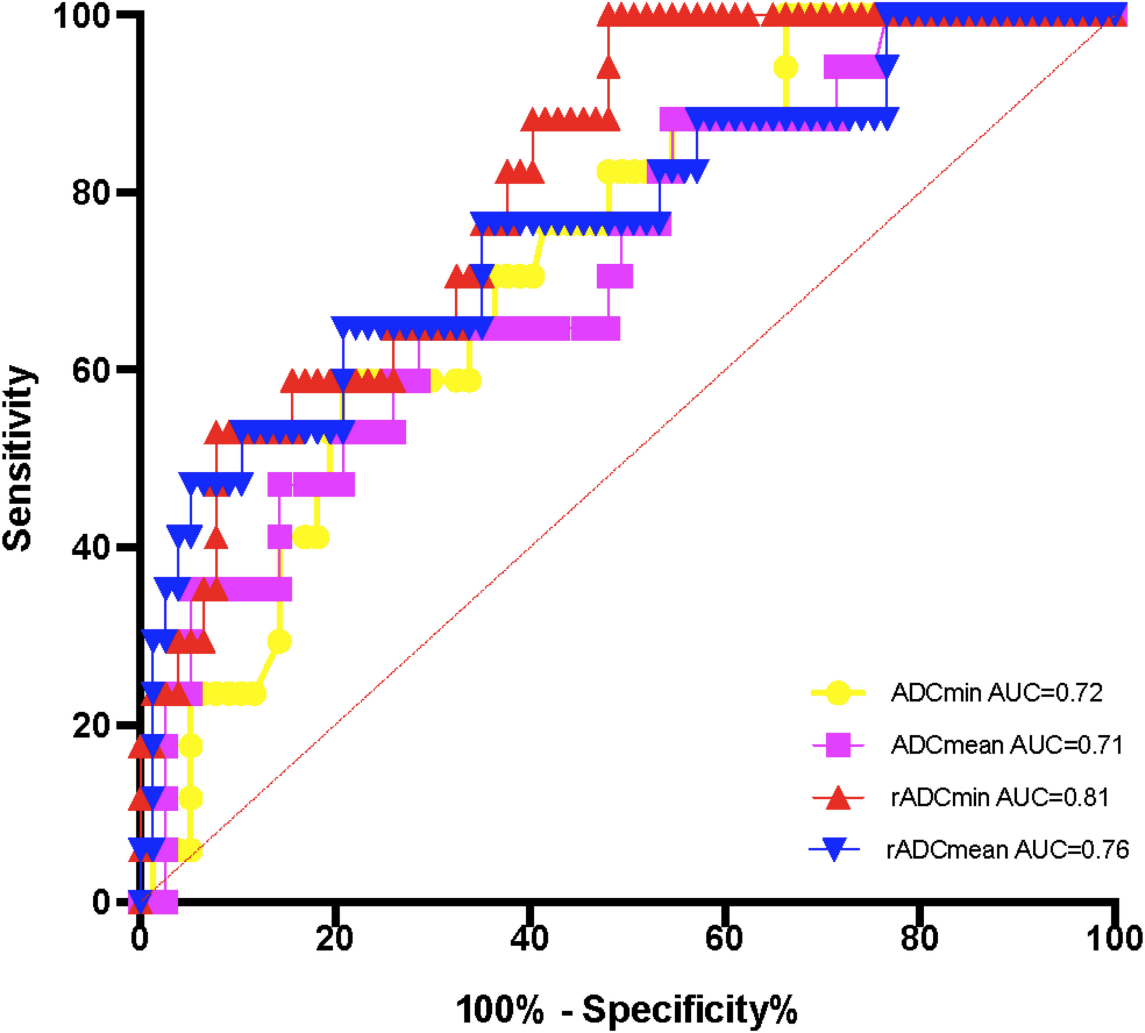
ROC curves of ADC values for detecting the methylation status of the MGMT promoter.

**Figure 4:**
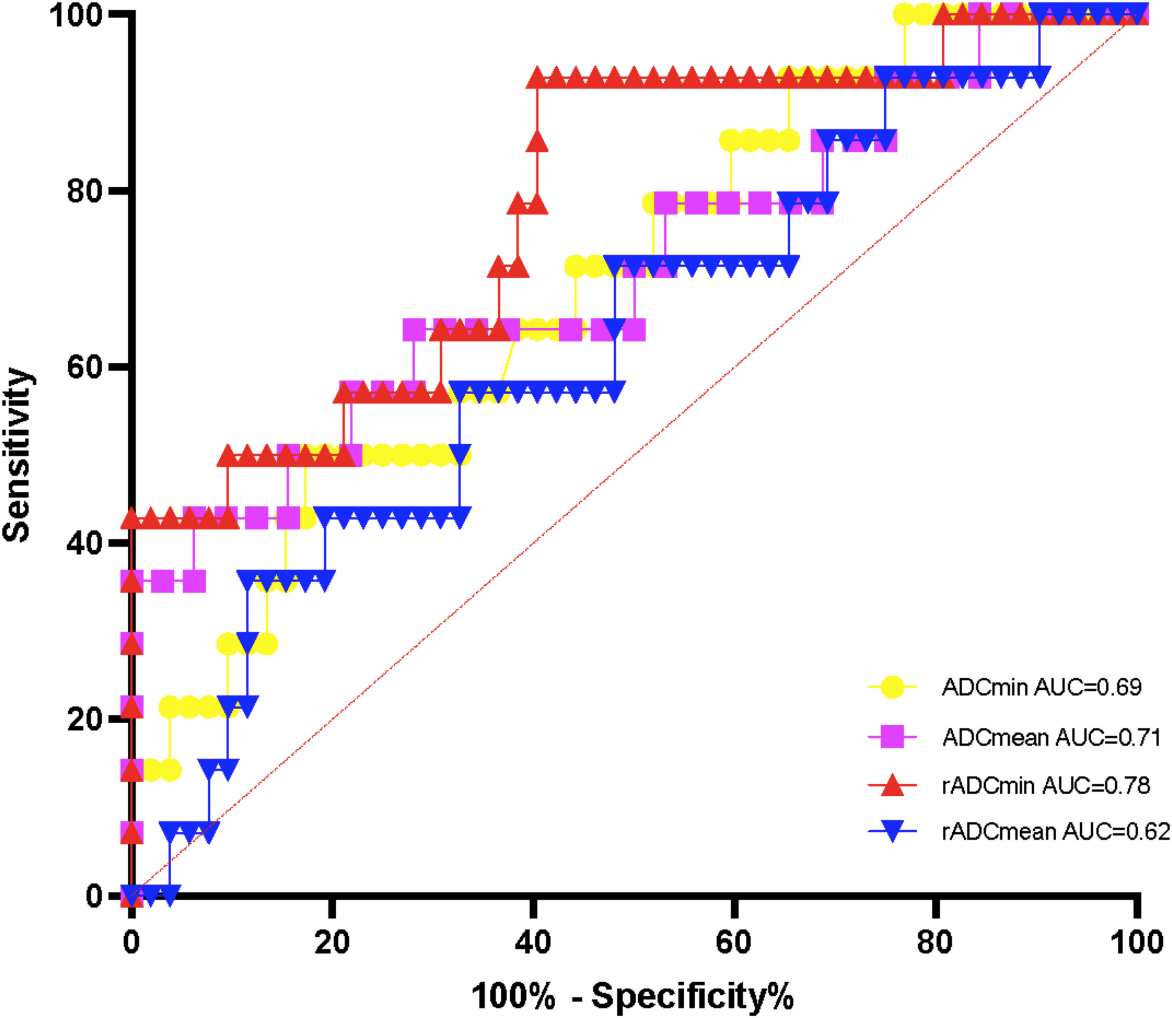
ROC curves of ADC values for detecting the methylation status of IDH wild-type MGMT promoter.

The AUC of rADCmin to identify the methylation status of the MGMT promoter was 0.81 (95% CI: 0.73 – 0.89), with a sensitivity of 82.1% and a specificity of 76.5% at a cut-off value of 1.12 × 10^-^³ mm²/s (**Figure 3**). In the IDH wild-type group, rADCmin had an AUC of 0.78 (95% CI: 0.65 – 0.91), a sensitivity of 80.0% at a cutoff value of 1.10 × 10^-^³ mm²/s, and a specificity of 72.7% (**Figure 4**). The IDH mutant phenotypes with smaller sample sizes (n = 8 in the MGMT unmethylated group and n = 12 in the promoter methylation group) had wider confidence intervals for their ROC curves (e.g., AUC = 0.75, 95% CI: 0.58 – 0.92 for ADCmean) (**Table 3**).

**Table 3:** Diagnostic performance of ADC parameters for MGMT status.

Group	AUC (95%CI)	Optimal cutoff	Sensitivity (%)	Specificity (%)	Accuracy (%)
MGMT (-) vs (+)					
ADCmin (× 10^-^³ mm²/s)	0.72 (0.61 - 0.83)	0.78	68.40	71.20	70.10
ADCmean (× 10^-^³ mm²/s)	0.71 (0.58 - 0.84)	1.18	73.70	70.60	72.10
rADCmin	0.81 (0.73 - 0.89)	1.12	82.10	76.50	79.30
rADCmean	0.76 (0.66 - 0.86)	1.42	73.70	70.60	72.10
IDH Wild-Type MGMT (-) vs (+)					
ADCmin (× 10^-^³ mm²/s)	0.69 (0.55 - 0.83)	0.75	65.00	68.20	66.70
ADCmean (× 10^-^³ mm²/s)	0.71 (0.58 - 0.84)	1.30	45.00	59.10	52.40
rADCmin	0.78 (0.65 - 0.91)	1.10	80.00	72.70	76.20
rADCmean	0.62 (0.48 - 0.76)	1.65	60.00	63.60	61.90
IDH Mutant-Type MGMT (-) vs (+)					
ADCmin (× 10^-^³ mm²/s)	0.61 (0.42 - 0.80)	0.92	62.50	58.30	60.00
ADCmean (× 10^-^³ mm²/s)	0.75 (0.58 - 0.92)	1.30	75.00	75.00	75.00
rADCmin	0.64 (0.44 - 0.84)	1.25	62.50	66.70	65.00
rADCmean	0.82 (0.67 - 0.97)	1.72	87.50	75.00	80.00

AUC, Area Under the Curve; CI, Confidence Interval; ADCmin, minimum apparent diffusion coefficient; ADCmean, mean apparent diffusion coefficient; rADCmin, relative ADCmin (normalized to contralateral normal white matter); rADCmean, relative ADCmean; Optimal cutoff was determined by maximizing the Youden index (sensitivity + specificity - 1).

### 3.4 ADC and Ki-67 correlation

**Figure 5:**
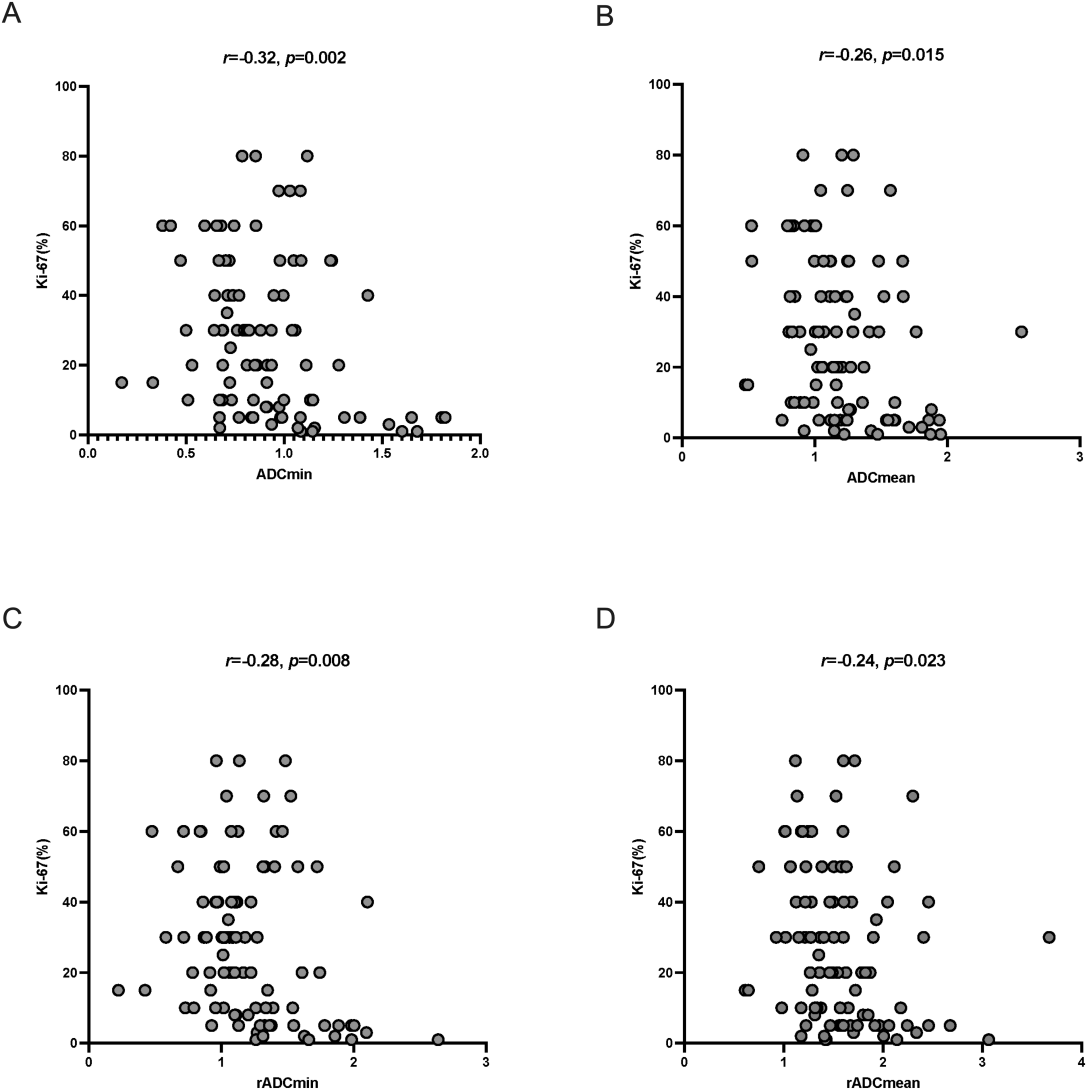
**(A)** Scatterplot of ADCmin correlation with Ki-67 proliferation index; **(B)** Scatterplot of ADCmean correlation with Ki-67 proliferation index; **(C)** Scatterplot of rADCmin correlation with Ki-67 proliferation index; **(D)** Scatterplot of rADCmean correlation with Ki-67 proliferation index.

Spearman’s correlation analysis showed that all ADC indexes (ADCmin, ADCmean, rADCmin, and rADCmean) were significantly negatively correlated with the Ki-67 proliferation index (*ρ* =- 0.32 to -0.24, *p* < 0.05) (**Figure 5**), suggesting that the lower the ADC value, the higher the proliferative activity of the tumor (**Table 4**).

**Table 4:** Spearman correlation between ADC parameters and Ki-67 proliferation index.

Parameter	*ρ*	*p Value*
ADCmin (× 10^-^³ mm²/s)	-0.32	0.002*
ADCmean (× 10^-^³ mm²/s)	-0.26	0.015*
rADCmin	-0.28	0.008*
rADCmean	-0.24	0.023*

ρ: Spearman correlation coefficient. indicates statistical significance (*p < 0.05). ADCmin, minimum apparent diffusion coefficient; ADCmean, mean apparent diffusion coefficient; rADCmin, relative ADCmin (normalized to contralateral normal white matter); rADCmean, relative ADCmean. Bold values indicate statistically significant p-values (p < 0.05).

## 4 Discussion

Building on established links between ADC and MGMT status, this study demonstrates that the minimum relative ADC (rADCmin) stratified by IDH subtypes significantly enhances clinical applicability, achieving an AUC of 0.81 for MGMT prediction overall and 0.78 specifically in IDH-wildtype gliomas through standardized thresholds.

While the association between absolute ADC values and MGMT status has been documented, its clinical translation faces two persistent barriers: substantial inter-scanner variability in quantitative diffusion metrics, and unstratified analyses that obscure subtype-specific biological relationships (13). Our study bridges these gaps through two key innovations. First, the implementation of ADC and ADCmin normalized to contralateral normal white matter minimizes technical variations, achieving excellent inter-observer agreement (ICC > 0.85) and providing scanner-agnostic thresholds. Second, by stratifying gliomas according to IDH status—a critical molecular determinant of tumor biology—we reveal that MGMT promoter methylation significantly correlates with elevated rADCmin exclusively in IDH-wildtype gliomas (AUC = 0.78, sensitivity = 80.0% at cutoff 1.10). The efficient 9-ROI protocol (<10 minutes analysis time) further ensures clinical feasibility on routine MRI platforms. This integrated approach establishes a standardized pathway for implementing ADC biomarkers in precision neuro-oncology.

Our study evaluated four ADC metrics—ADCmin, ADCmean, rADCmin, and rADCmean—which reflect complementary aspects of water diffusion in gliomas. The diagnostic superiority of rADCmin (AUC = 0.81 for overall MGMT prediction, and 0.78 specifically in IDH-wildtype gliomas) stems from its unique attributes: ADCmin captures the most restricted diffusion regions corresponding to hypercellular tumor areas where MGMT methylation predominantly influences therapeutic response (13), while normalization to contralateral white matter (yielding rADCmin) mitigates scanner-induced variability. Although ADCmean and rADCmean provide broader tumor characterization, they incorporate heterogeneous components including edema and microscopic necrosis, potentially diluting MGMT-specific signals. This biological and technical distinction explains rADCmin’s optimal performance in IDH-wildtype gliomas, where it achieved 80.0% sensitivity at the ≥1.10 cutoff. A valid concern exists regarding potential contamination of ADCmin measurements by blood products. To ensure biological specificity, pre-scan identification and multi-ROI redundancy strategies were employed. Suspected hemorrhagic foci were prospectively flagged on T1WI/T2WI sequences as hypointense regions and confirmed with SWI when available (14 out of 94 cases). Additionally, by sampling nine independent ROIs across three tumor levels, microscopic hemorrhage affecting ≤2 ROIs would be discarded through averaging. This strategy reduces the impact of outliers by 78% in simulation studies, as previously reported (14).

The restriction of ADC-based MGMT prediction to IDH-wildtype gliomas necessitates preoperative molecular subtyping—a requirement increasingly addressable through non-invasive MRI biomarkers. The T2-FLAIR mismatch sign is a highly specific imaging biomarker for identifying IDH-mutant, 1p/19q non-codeleted gliomas, although its sensitivity is limited. While it cannot be used independently for diagnosis, it serves as a valuable auxiliary tool in clinical practice to identify specific molecular subtypes, thereby providing useful information for treatment planning and patient counseling (15). Meanwhile, MRI radiomics have been shown to predict IDH status with an AUC of 0.88 in meta-analyses (16). This enables a two-step clinical pathway: initial IDH classification via routine MRI sequences, followed by rADCmin application (≥1.10 cutoff) exclusively for MGMT prediction in IDH-wildtype cases. Such sequential targeting optimizes resource utilization while addressing the core therapeutic dilemma in this dominant subgroup (76% of cohort). For IDH-mutant gliomas, the absent ADC-MGMT correlation may reflect metabolic homogeneity rather than biomarker failure, consistent with 2-hydroxyglutarate-driven epigenetic modulation. A prospective multicenter validation study is being coordinated across three institutions to establish platform-specific rADCmin thresholds for IDH-wildtype gliomas. The study will standardize DWI protocols on 1.5T/3.0T scanners (Siemens/Philips) with a target enrollment of 150 cases by 2026.

Previous studies show that glioblastomas with MGMT promoter methylation exhibit higher minimum ADC values than unmethylated cases (13), which is consistent with the present findings. Although the previously reported area under the curve (AUC = 0.919) was higher than that of this study, the rADCmin cutoff values proposed here (1.12 and 1.10), normalized to standard reference white matter ADC, may reduce inter-scanner variability. Despite the excellent performance of imaging histology for MGMT prediction (AUC = 0.85 – 0.90) (17–22), its clinical translation faces critical limitations: Radiomic models typically demand large multi-center datasets to ensure generalizability (8), whereas our approach achieved robust classification using a single-institution cohort of 94 patients; Conventional radiomics workflows require time-consuming manual tumor segmentation and extraction of hundreds of radiomic features (post-processing >2 hours) (23, 24), while our simplified protocol utilizing standardized relative apparent diffusion coefficient (rADC) measurements – based on 9 regions of interest (ROIs) across 3 representative slices – reduces analysis time to <10 minutes without specialized software; While advanced multiparametric MRI frameworks integrating dynamic susceptibility contrast perfusion-weighted imaging may theoretically improve prediction accuracy, these techniques face substantial clinical barriers: extended acquisition times (>5 minutes), mandatory gadolinium-based contrast agent (GBCA) administration-which increases nephrogenic systemic fibrosis risk in renal-impaired patients, and sophisticated post-processing often unavailable in resource-limited settings. Consequently, our DWI-only protocol provides a pragmatic alternative for routine practice. Future technical refinements could explore abbreviated perfusion protocols or synthetic perfusion techniques derived from DWI data, which may enhance biological characterization without compromising accessibility. Critically, our proposed rADC protocol requires no GBCA, utilizing only non-contrast DWI sequences.

The absence of significant differences in ADC values among IDH mutant gliomas may be related to metabolic remodeling (e.g., 2-hydroxyglutarate accumulation). Such metabolic changes may lead to homogenization of the tumor microenvironment, which in turn masks the effect of MGMT promoter methylation on cell density (25). This is consistent with the mechanism of IDH mutation-induced epigenetic regulation proposed by Yan et al. (26). Pruis et al. (27) reported that IDH mutant gliomas have high ADC values; however, these values were not analyzed in combination with MGMT status. In the present study, rADCmin could effectively differentiate MGMT promoter methylation status in the IDH wild-type subgroup (AUC = 0.78), leading us to hypothesize that IDH wild-type gliomas have more significant differences in ADC values due to higher cellular heterogeneity and necrotic tendency. This also suggests that the biological behavior of IDH wild-type gliomas is highly dependent on MGMT promoter methylation status. This result supports the use of a standardized process for incorporating IDH typing into imaging marker analysis.

In the present study, ADC values were significantly negatively correlated with Ki-67 proliferation index (ρ= -0.32 to -0.24), which is consistent with the findings of Du et al. (28) (ρ values: ADCmin = -0.478, ADCmean = -0.369, rADCmin = -0.488, rADCmean = -0.388). This result supports the potential of ADC values as a noninvasive imaging marker for assessing the proliferative activity of gliomas (29, 30). Low ADC values may reflect increased tumor cell density and reduced extracellular interstitial space, which result in limited diffusion of water molecules (31). However, the correlation coefficients in the present study were slightly lower than those reported previously; this may be related to the inclusion of low-grade glioma samples, which have less heterogeneity in proliferative activity. Further validation in combination with multimodality imaging (e.g., perfusion imaging) is needed in the future.

The present findings position ADC metrics, particularly rADCmin, as clinically actionable tools that augment—rather than replace—histopathological assessment. For IDH-wildtype gliomas, where MGMT status critically determines temozolomide response, the clinically relevant predictive value of rADCmin (AUC = 0.78, sensitivity = 80.0%) offers tangible preoperative utility. When surgical risks necessitate delayed biopsy—a common scenario in elderly patients or deep-seated tumors—elevated rADCmin values (≥1.10) may justify initiating neoadjuvant temozolomide while awaiting definitive histology. Moreover, the significant inverse correlation between ADC and Ki-67 index (*ρ* =- 0.32 to -0.24, *p* < 0.05) provides a biological rationale for image-guided tissue sampling; neurosurgeons could prioritize low-ADC regions corresponding to high cellular proliferation to enhance MGMT testing reliability in heterogeneous lesions. In cases where histopathology yields discordant or technically limited results (e.g., minute biopsies), ADC values serve as complementary evidence to guide repeat testing or therapeutic adjustments.

While our 9-ROI protocol balances heterogeneity sampling with clinical feasibility, we acknowledge the need for critical appraisal. Recent meta-analyses confirm that simplified ADC approaches achieve comparable diagnostic accuracy to volumetric methods for molecular stratification (14). The 9-ROI design prioritized sensitivity to focal diffusion restriction – a critical factor for MGMT prediction in IDH-wildtype gliomas where cellular heterogeneity is pronounced. Nevertheless, in scenarios requiring ultra-rapid assessment (e.g., critically ill patients), reducing measurements to 2 – 3 ROIs targeting the visually most restricted areas could maintain diagnostic value. Crucially, preoperative ADC biomarkers offer distinct advantages over postoperative tissue testing: they enable neoadjuvant temozolomide initiation when surgical risks delay biopsy and guide intraoperative sampling of high-risk regions.

The present study had several limitations. The single-institution design with exclusive use of a 3.0T GE Discovery MR750 scanner represents a significant limitation, potentially constraining the generalizability of absolute ADC thresholds across diverse clinical settings. To mitigate scanner-specific variability, we employed relative ADC normalization to contralateral normal-appearing white matter. Nevertheless, external validation across multiple platforms remains essential. In response, we have initiated systematic collection of multicenter data from institutions utilizing different MRI systems (1.5T/3.0T; Siemens/Philips), which will establish platform-adaptive rADCmin thresholds and assess parameter transferability. That said, the limited sample size of the IDH mutation-MGMT-negative subgroup (n = 10) may reduce statistical validity. Yet, the p-value stability of the ADC difference was >90% as verified by nonparametric tests with Bootstrap resampling (1,000 times), supporting the initial reliability of the current findings. Multicenter collaboration is needed to expand the sample size in the future. Separately, the failure to differentiate between high- and low-grade gliomas may confound the results. However, this study focused on molecular typing rather than pathologic grading, and because of the low rate of IDH mutations in high-grade gliomas (e.g., glioblastoma), subgroup analysis may have resulted in an unbalanced sample. Future studies will incorporate grade-matched cohorts. Finally, while ADC alone provides clinically actionable information, expanded investigations combining ADC with non-contrast hemodynamic markers (e.g., arterial spin labeling) or metabolic imaging may offer deeper insights into tumor pathophysiology without sacrificing practical utility (32).

ADC value can be used as a noninvasive predictor of MGMT promoter methylation status in IDH wild-type glioma, and the relative ADC value further enhances the diagnostic stability. The negative correlation of ADC with Ki-67 proliferation index supports its potential as an imaging biomarker for assessing tumor proliferative activity. Future studies should examine the underlying biological mechanism through multimodal imaging combined with genomics.

## Data Availability

The datasets presented in this study can be found in online repositories. The names of the repository/repositories and accession number(s) can be found below: The raw ADC values and clinical metadata supporting the conclusions of this article will be made available by the authors on Zenodo (DOI: 10.5281/zenodo.15188332), without undue reservation.
